# Alarming Signal from the Ear: Tinnitus, a Possible Epiphenomenon of Silent Hyperlipidaemia

**DOI:** 10.3390/biomedicines12122837

**Published:** 2024-12-13

**Authors:** Zsuzsanna Piros, Kristóf Kispál, Réka Szekeres, Barbara Takács, Rita Kiss, Adrienn Mónika Szabó, Dóra Ujvárosy, Zoltán Szabó, Zoltán Szilvássy, Rudolf Gesztelyi, Béla Juhász

**Affiliations:** 1 Department of Otolaryngology and Head and Neck Surgery, University of Debrecen Clinical Centre, Nagyerdei St. 98, H-4032 Debrecen, Hungary; dr.piros.zsuzsanna@med.unideb.hu (Z.P.); kispal.kristof@med.unideb.hu (K.K.); 2 Doctoral School of Nutrition and Food Sciences, University of Debrecen, H-4032 Debrecen, Hungary; 3 Department of Pharmacology and Pharmacotherapy, Faculty of Medicine, University of Debrecen, Nagyerdei St. 98, H-4032 Debrecen, Hungary; szekeres.reka@med.unideb.hu (R.S.); takacs.barbara@pharm.unideb.hu (B.T.); kiss.rita@med.unideb.hu (R.K.); szilvassy.zoltan@med.unideb.hu (Z.S.); gesztelyi.rudolf@pharm.unideb.hu (R.G.); 4 Department of Internal Medicine (Building C), University of Debrecen Clinical Centre, Moricz Zsigmond krt. 22, H-4032 Debrecen, Hungary; szabo.adrienn@med.unideb.hu; 5 Department of Emergency Medicine, University of Debrecen Clinical Centre, Nagyerdei St. 98, H-4032 Debrecen, Hungary; ujvarosy.dora@med.unideb.hu (D.U.); szabo.zoltan@med.unideb.hu (Z.S.)

**Keywords:** tinnitus, atherosclerotic cardiovascular disease, chronic subjective idiopathic tinnitus, hypercholesterolaemia, hypertriglyceridemia, hyperlipidaemia, dyslipidaemia, atherosclerosis, low-density lipoprotein, high-density lipoprotein

## Abstract

**Background/Objectives:** Over the past few decades, many studies have been conducted to explore the link between tinnitus and lipid metabolism, yielding inconsistent results. In our current study, we compared the prevalence of various lipid metabolism abnormalities in patients with chronic subjective idiopathic tinnitus (CSIT) to the official prevalence data of dyslipidaemia in the general adult Hungarian population. To counteract the distorting effect of the co-increase in both conditions with age, we also examined this relationship by age groups. **Methods:** A total of 231 adult patients, suffering from CSIT, who underwent rheological treatment at the Department of Otorhinolaryngology and Head and Neck Surgery of the Clinical Center (University of Debrecen, Debrecen, Hungary), were involved in a retrospective study. Total cholesterol, serum triglycerides, LDL-C, ApoB, HDL-C, ApoA, and Lp(a) were utilized. **Results:** For the aggregated data, we found that the occurrence of dyslipidaemia among our patients (78.35%) significantly exceeded the corresponding official data about the occurrence of dyslipidaemia in the whole adult Hungarian population (16.51%). This finding was supported by our age-specific approach and the results imply an association between CSIT and dyslipidaemia. This finding suggests an association between CSIT and dyslipidaemia. **Conclusions:** Considering the relationship between CSIT and dyslipidaemia suggested by our results, tinnitus could be an indicator of dyslipidaemia, even at a young age. Therefore, careful investigation of each tinnitus patient, along with a lipidological evaluation for those with CSIT, may be recommended. This approach could lower mortality from lipid-related diseases, particularly atherosclerosis and its complications, by serving as the first line of defence against a harmful and life-threatening lipid-related conditions.

## 1. Introduction

Tinnitus is the perception of sound that occurs without external acoustic stimuli [1,2]. It affects 35–40% of people during their lifetime [3]. About 20 years ago, the prevalence of this condition was around 13% to 17% among the total adult population [4], a figure that has roughly doubled since that time [5]. Approximately 20% of individuals affected by tinnitus find it bothersome enough to seek medical attention, whereas 1–2% report suffering an all-day, excruciating, and intolerable form of tinnitus that significantly impacts their quality of life [6].

Tinnitus can be categorized based on several criteria, as follows: temporality (acute or chronic), detectability of the sound (objective or subjective), and underlying aetiology (primary idiopathic or secondary tinnitus). Tinnitus that persists for more than six months is classified as chronic. Objective tinnitus is defined as sounds perceived by the patient without an external sound source, which can also be detected by an observer. In such cases, the sound originates from within the patient’s body and is often identifiable. These are classified as secondary forms of tinnitus. In contrast, subjective tinnitus is perceived only by the patient in the absence of an external sound source [7]. Secondary tinnitus develops when recognized etiological factors with a confirmed or likely causal link to tinnitus are present. Idiopathic/primary tinnitus is an exclusionary diagnosis, requiring the confirmation or elimination of the conditions associated with tinnitus. When there is no identifiable underlying etiological factor, subjective tinnitus is diagnosed. Subjective idiopathic tinnitus may be associated with sensorineural hearing loss (in approximately half of the cases). If so, it is known as sensorineural tinnitus, a type of primary tinnitus. If not, the condition is recognized as normal-hearing tinnitus [8]. Currently, there is no generally accepted standard treatment method for tinnitus, and given the multifaceted nature of the symptoms, there probably will not be a single one-size-fits-all solution. Thus, its treatment remains unresolved. Most patients must adapt and learn to manage this possibly excruciating and distressing symptom. Given that tinnitus exacerbates distress and, in unattended cases, can lead to psychiatric comorbidities such as depression and anxiety, its impact on mental health is substantial. Approximately 20% of patients with tinnitus exhibit detectable psychiatric comorbidities. The critical role of depressive symptoms in chronic tinnitus has been well-documented and is often explained by a “vicious cycle”, where the depressive symptoms not only facilitate the persistence of tinnitus as a chronic condition but also act as a maintenance factor, perpetuating the condition. Consequently, reducing the tinnitus handicap is essential for mitigating distress and preventing the development of comorbidities [9]. The WHO characterizes tinnitus as a nonspecific symptom rather than a distinct clinical condition. However, some authors propose differentiating between “tinnitus” and “tinnitus disorder” to more effectively acknowledge the latter as a primary health issue. The difference between the two is that if the patient suffers from the psychological burden of tinnitus, then the diagnosis is tinnitus disorder, but if not, then it is tinnitus, which can be graded by the Tinnitus Handicap Inventory [10].

Various factors can play a role in the development and maintenance of tinnitus. However, its aetiology and pathophysiology are not completely known. The accepted theories have two fundamental approaches, giving explanations based on cochlear and central origins. The former considers tinnitus as sensory (peripheral) in origin, attributing it to damage and dysfunctional processes within the inner ear. The latter approach attributes tinnitus primarily to the dysfunctional operation of the central nervous system (central origin). Emotional networks and their rearrangement with the auditory system has been connected to troublesome tinnitus [11,12,13]. Overall, changes in the activity of extensive regions of the cortex and subcortical structures have been identified in relation to tinnitus [14].

Tinnitus has been associated with various comorbidities and medical conditions. These may include factors that are correlated with tinnitus or those that trigger or exacerbate it. It is well-established that vascular abnormalities, hypertension, thyroid disorders, and certain medications are linked to tinnitus. Recent studies have also investigated the relationship between tinnitus and lipid metabolism, as well as dyslipidaemias [15,16,17,18]. Dyslipidaemia, especially elevated TC and LDL levels, is associated with an increased risk of cardiovascular diseases (CVD), primarily atherosclerotic cardiovascular disease (ASCVD), serving as a well-known risk factor. Additionally, they are linked to other conditions, such as hypertriglyceridemia with acute pancreatitis and non-alcoholic fatty liver disease. The significance of the relationship between dyslipidaemia, particularly elevated LDL-C, and ASCVD lies in the fact that ASCVD is the leading cause of death globally, responsible for most deaths worldwide [19].

Over the past three decades, the prevalence of dyslipidaemia has shown an increase, imposing a significant health burden globally. Moreover, it is affecting more young adults (aged 30–40), which is alarming as it is associated with an elevated risk of coronary diseases in later stages of life. According to the research data, young adults with elevated TC have a fivefold higher likelihood of developing coronary diseases and a ninefold higher risk of acute myocardial infarction than their peers with normal TC levels at the same age. The global increase in the prevalence of dyslipidaemia can be credited to a combination of various factors, such as urbanization and globalization, changes in dietary patterns, a rise in the consumption of processed foods rich in fats and sugars, a decrease in physical activity, and an increase in obesity rates. Along with these lifestyle changes, factors such as socioeconomic conditions, access to healthcare, and the aging population are important contributors to the global increase in the prevalence of lipid abnormalities worldwide.

The link between tinnitus and lipid metabolism has been investigated by several researchers. Some data confirm the presence of dyslipidaemia in tinnitus [16,20,21], while other data contradict it [22,23]. Concurrently, various research findings have suggested that changes in lipid metabolism can harm both the cochlea and the stria vascularis through multiple mechanisms. According to some animal studies, hyperlipidaemia diminishes the electromotility of hair cells due to lipid accumulation [24], while others find electromicroscopical structural alteration (e.g., vacuolar degeneration) in the cochlea [25,26,27]. Furthermore, the inner ear functions as an “end-organ” concerning its circulation, where end arteries ensure its supply. Thus, lipid accumulation and atherosclerotic processes, similar to those occurring in the cardiac muscle, may ultimately result in oxidative damage to the stria vascularis and hair cells [20,21,28,29]. According to other hypotheses, disruptions in lipid metabolism might initiate or exacerbate inflammatory processes. Inflammatory mediators, such as cytokines, could impact both the inner ear and neural structures associated with hearing, and cause tinnitus [1,30]. Several authors have proposed that the origin of tinnitus lies not in the inner ear, but in the central nervous system (central tinnitus theories). Dyslipidaemia may contribute to the onset or persistence of tinnitus by damaging the cerebrovascular system via similar mechanisms as those affecting the systemic circulation [31]. Despite numerous questions and conflicting results, an increasing body of research has corroborated the association between lipid metabolism and tinnitus.

Given that dyslipidaemia itself is symptomless, only regular laboratory tests provide an opportunity for early detection and secondary prevention [32]. At the same time, the widespread use of statins and their increasing availability contribute to the reduction in the disease burden associated with ASCVD. However, a self-evident requirement for effective statin treatment is also the recognition of otherwise asymptomatic dyslipidaemia.

If the link between tinnitus and dyslipidaemia is confirmed, tinnitus could serve as an early alarming sign, drawing attention to the underlying lipid metabolism disorder. This offers an opportunity for the early secondary prevention of both dyslipidaemia and the other serious consequences associated with this silent killer phenomenon.

## 2. Materials and Methods

### 2.1. Patients

We conducted a retrospective study involving 652 consecutive adult patients who underwent rheological treatment at the Department of Otorhinolaryngology and Head and Neck Surgery of the Clinical Center, University of Debrecen (CCUD), between 1 January 2022, and 1 May 2023, of which 231 patients were included in the retrospective analysis after the exclusion criteria were validated. This study was designed in accordance with the principles of the Declaration of Helsinki, and it was approved by the Regional Institutional Research Ethics Committee, Clinical Center, University of Debrecen (number: DE RKEB/IKEB 6480-2023).

**Figure 1:**
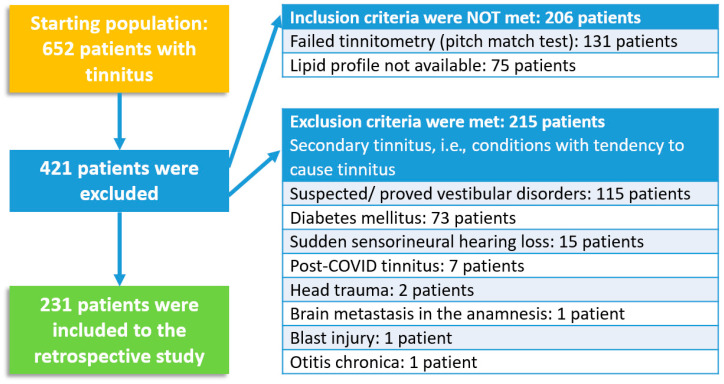
Flowchart of exclusion and inclusion criteria.

The inclusion criteria were as follows: (1) the diagnosis of chronic, idiopathic, subjective tinnitus (CSIT), no matter the presence of sensorineural hearing loss or laterality, and (2) an available lipid profile assessed at the Laboratory Medicine Institute of CCUD. The exclusion criteria were as follows: (1) every tinnitus proved to be secondary, i.e., conditions with tendency to cause tinnitus such as Ménière’s disease, otosclerosis, vestibular schwannoma (acoustic neurinoma), therapy using a drug with known ototoxicity, sudden hearing loss, conductive hearing loss, or mixed hearing loss, whether confirmed by audiometric testing or suggested by tympanometric results (As-, Ad-, B-, C-type tympanograms), previous head trauma, current ear infections (otitis externa, otitis media, chronic middle ear effusion), ear abnormalities (e.g., eardrum perforation or retraction), or a history of neuro-otological surgery. Hypertension was defined as patients having a systolic blood pressure ≥ 140 mm Hg and/or a diastolic blood pressure ≥ 90 mm Hg, or those who reported taking high blood pressure medication. Controlled hypertension was defined as a systolic blood pressure < 140 mm Hg and a diastolic blood pressure < 90 mm Hg among patients with hypertension). (2) the presence of systemic diseases (especially malignancies, diabetes, endocrine disorders), autoimmune diseases, neurological or psychiatric diseases; (3) a lipid profile being incomplete or assessed elsewhere were excluded from this study (to ensure the homogeneity of the methodological background) (Figure 1).

The diagnosis of CSIT was confirmed through a comprehensive otorhinolaryngological (ORL) physical examination (including otoscopy and otomicroscopy), audiological, neurological, dental, temporomandibular joint, and internal medicine specialty examinations, along with imaging studies/head MRI (if it is contraindicated or the patient refuses the examination, a head CT scan, inner ear HR-CT, or inner ear MRI.

### 2.2. Examinations

The chronic nature of tinnitus (lasting more than 6 months) was determined from the clinical history (anamnestic data). Auscultation during the ORL physical examination was used to rule out the objective tinnitus. The audiometric test battery included a pure-tone audiometry, stapedius reflex measurements, tympanometry, and otoacoustic emissions. Audiometric tests comprised air- and bone-conduction pure-tone thresholds from 125 Hz to 8000 Hz, reflex measurements, tympanometry, and tinnitometry (pitch match test). Patients having abnormal tympanometry (other than a type A curve) were excluded. Tinnitometry was utilized to determine the frequency and intensity of tinnitus, thereby confirming the presence of a subjective tinnitus. Cases with tinnitus that could not be measured by tinnitometry (tinnitus pitch match and loudness analysis) were also excluded. If a retrocochlear origin or another central cause was suspected (particularly in cases of unilateral tinnitus or those associated with unilateral or asymmetric sensorineural hearing loss), an MRI examination was performed. In cases of associated dizziness or balance disorders, an otoneurological examination was performed to rule out vestibular disorders (benign paroxysmal positional vertigo (BPPV), labyrinthitis, or vestibular neuritis, Ménière’s disease, and secondary endolymphatic hydrops, superior semicircular canal dehiscence, vestibular schwannoma, perilymph fistula, enlarged vestibular aqueduct, migraine-associated vertigo, etc.). Neurotological examination included the following: detection of spontaneous nystagmus, nystagmus with fixation suppression (using Bartels goggles), saccadic movements, Barany test, Romberg test, Unterberger’s test, video head impulse test (vHIT), static posturography, and in cases of suspected Ménière’s disease, caloric stimulation testing was also conducted.

Examination protocol

1. ORL physical examination
  - Case history
  - Clinical examination
    - Otoscopy, otomicroscopy;
    - Auscultation (for objective tinnitus);
    - Examination and exclusion of secondary tinnitus;
    - Other examinations based on the nature and type of tinnitus (the tinnitus examination protocol based on the nature of tinnitus);
    - Laboratory (blood count, general, thyroid hormone, etc.).
2. Audiology
  - Subjective and objective hearing tests/pure-tone audiometry (see: Table 1), tinnitometry, impedance tests, transient evoked otoacoustic emissions (TE OAE), distortion product otoacoustic emissions (DP OAE)/;
  - Special examinations in the case of tinnitus patients, as follows: high-frequency audiometry, tinnitometry, determination of discomfort threshold.
3. Neurotology
  - Primarily in case of associated dizziness complaints;
  - Suspected acoustic neurinoma.

Considering the previously specified criteria, 231 out of the initial 654 patients were included in the retrospective study. Patients were categorized into seven subgroups based on the age classification of the Hungarian Central Statistical Office (HCSO, in Hungarian: Központi Statisztikai Hivatal, KSH), as follows: 19–24 years old, 25–34 years old, 35–44 years old, 45–54 years old, 55–64 years old, 65–74 years old, and 75 years or older.

The investigated serum lipid parameters (and their levels considered to be pathological) were the following: total cholesterol (>5.20 mmol/L), triglycerides (>1.7 mmol/L), LDL-C (>3.40 mmol/L), ApoB (>1 g/L), HDL-C (<1.30 mmol/L), ApoA (<1.15 g/L), and Lp(a) (>300 mg/L). If the value of any lipid parameter was found to be pathological, the patient was put into the dyslipidaemic group; otherwise, the patient was placed in the non-dyslipidaemic group. During the assessment of patient history, previously diagnosed dyslipidaemias by general practitioners, internists, or lipidologists were also considered, forming a group classified as dyslipidaemic within the study cohort. All patients identified as dyslipidaemic through this process were included in this study, provided they met the other inclusion criteria. This was the case even if they were receiving treatment, whether pharmacological therapy or lifestyle modifications. Irrespective of their current laboratory parameters, they were classified in the dyslipidaemic group based on their documented diagnosis and treatment history for dyslipidaemia.

### 2.3. Statistical Analysis

Normality of the data sets was investigated with the D’Agostino–Pearson test. The correlation between the main lipid parameters (total cholesterol, triglyceride, LDL-C, HDL-C, Lp(a)) and the patients’ age was determined with Pearson’s method (if both data sets followed Gaussian distribution) or with Spearman’s method (if not). Precision of the correlation was characterized by the 95% confidence interval (CI) of the correlation coefficient (r). For the sake of presentation, linear regression was also performed when the correlation was significant (to highlight the presence of the significant correlation). Precision of the regression was visualized by the 95% confidence bands around the best-fit straight line. These data were also evaluated after breaking down them by gender.

To compare the occurrence of dyslipidaemia among our patients and the whole Hungarian population (obtained from HCSO), Fisher’s exact test was performed on the two data sets as a whole and both were broken down by age. The age-specific frequency data of our patients were normalized to 10,000 individuals to align with the data provided by HCSO in its latest Health Overview (published in 2019).

For the statistical analysis, GraphPad Prism 9.5.1 for Windows (GraphPad Software Inc., La Jolla, CA, USA) was used. Differences were considered significant at *p* < 0.05. Data are presented in a form as indicated.

## 3. Results

During our retrospective analysis, 231 out of 652 patients met the inclusion criteria, with a mean age of 59.68 years (±14.67). The cohort consisted of 95 men and 136 women, reflecting a slight predominance of females. Notably, no significant gender dominance was observed in the age groups between 18 and 54 years; however, a female predominance became apparent in individuals aged 55 years and older. The main audiological parameters related to tinnitus of the 231 patients included are summarized in Table 1.

### 3.1. Occurrence of the Previously Known Dyslipidaemia Among Our Patients

A total of 18 patients (of 231; 7.8%) had previously known dyslipidaemia, 16 of whom received pharmacological treatment (statins) in addition to the recommended lifestyle changes. A total of 14 (of 18) still had an abnormal lipid profile at the time of the examination (despite the recommended dietary restrictions and drugs) (Table 2).

### 3.2. Serum Lipid Levels of Our Patients

#### 3.2.1. The Serum Triglyceride Level

The serum triglyceride level was increased in 92 patients (39.8%), and in an isolated form, in 11 cases (4.8%) (Table 3).

#### 3.2.2. The Serum Total Cholesterol Level

In turn, the serum total cholesterol level was elevated in 132 patients (57.1%), and its mean was above the normal level (5.37 ± 1.26 mmol/L) (Table 4).

#### 3.2.3. The Serum LDL-C Level

The serum LDL-C level was elevated in 97 patients (41.9%) (Table 5). In contrast to the aforementioned laboratory parameters, the mean LDL-C level was below the upper limit of the normal range (3.19 ± 1.04 mmol/L).

#### 3.2.4. The Serum ApoB Level

The serum ApoB level was available for 163 (70.6%) patients, of which it was higher than the normal in 76 cases (46.6%). Interestingly, the average serum level of ApoB, the primary apolipoprotein of LDL-C, was considerably above the normal value (7.09 ± 9.71 g/L) (Table 6).

#### 3.2.5. The Serum HDL-C Level

The serum HDL-C level decreased in 67 cases (29.0%), but its mean was above the minimal threshold value (1.50 ± 0.42 mmol/L) (Table 7).

#### 3.2.6. The Serum ApoA Level

The serum ApoA level was available for 170 patients (73.6%), 15 of which (8.8%) showed lower levels than the normal one. Isolated abnormality was detected in 2 patients (1.2%). Consistent with the average HDL level, the average serum level of ApoA, the primary apolipoprotein of HDL-C, was also above the threshold value (1.63 ± 0.67 g/L) (Table 8).

#### 3.2.7. The Serum Lp(a) Level

The serum Lp(a) level was available for 158 patients (68.4%), showing an elevated value in 32 cases (20.2%). Isolated increases in Lp(a) were found in 10 cases, and 2 of these patients received statin treatment. The mean serum Lp(a) level was below the threshold value (158 ± 324.33 mg/L) (Table 9).

### 3.3. Occurrence of Dyslipidaemia in Our Patients vs. in the Adult Hungarian Population

Overall, in our present investigation, pathological values of lipid parameters were found in 181 of 231 patients (78.3%) (Table 10). Additionally, a combined increase in triglyceride and Lp(a) levels (with normal values of the other lipid parameters) was seen in four patients, while a combined increase in the total cholesterol and Lp(a) levels was found in one patient.

**Figure 2:**
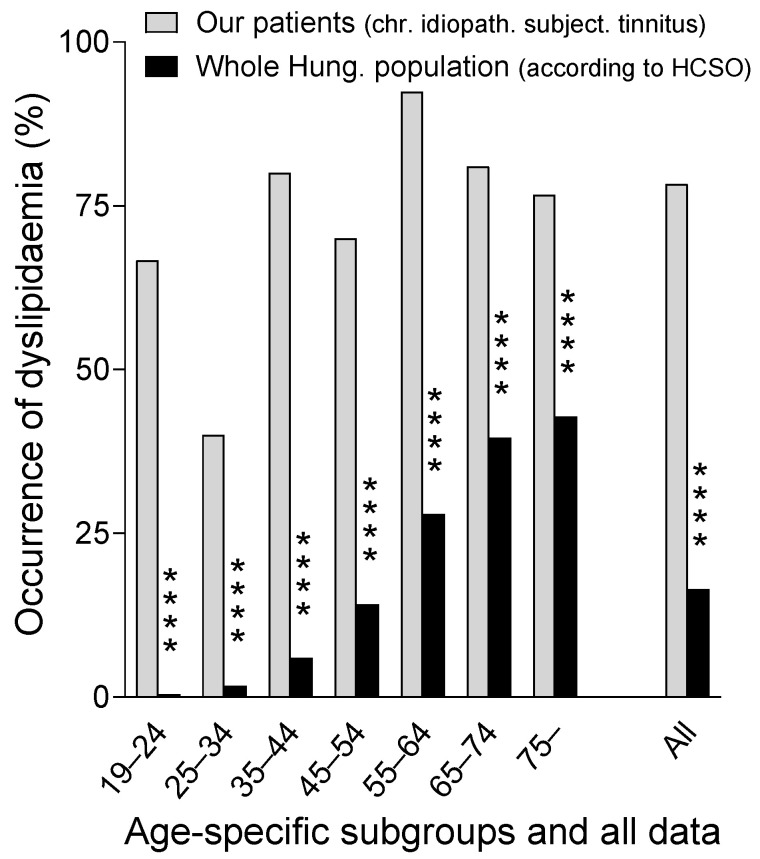
The occurrence of dyslipidaemia among our adult patients involved in this study (with chronic, idiopathic, and subjective tinnitus) and among the whole adult Hungarian population according to the official national statistical database (Hungarian Central Statistical Office: HCSO). Data were compared both in the age ranges used by HCSO and in aggregated form. ****: *p* < 0.0001 (provided by Fisher’s exact test).

We found that the occurrence of dyslipidaemia in our adult patients statistically significantly exceeded the corresponding HCSO data reporting the whole adult Hungarian population, for both the aggregated and age-specific data (Table 10, Figure 2). This finding suggests an association between the occurrence of chronic, idiopathic, subjective tinnitus, and dyslipidaemia. The link between chronic, idiopathic, subjective tinnitus, and dyslipidaemia may be a pathologic condition that increases the risk of both dyslipidaemia and chronic, idiopathic, and subjective tinnitus (independently or in connection with each other).

**Figure 3:**
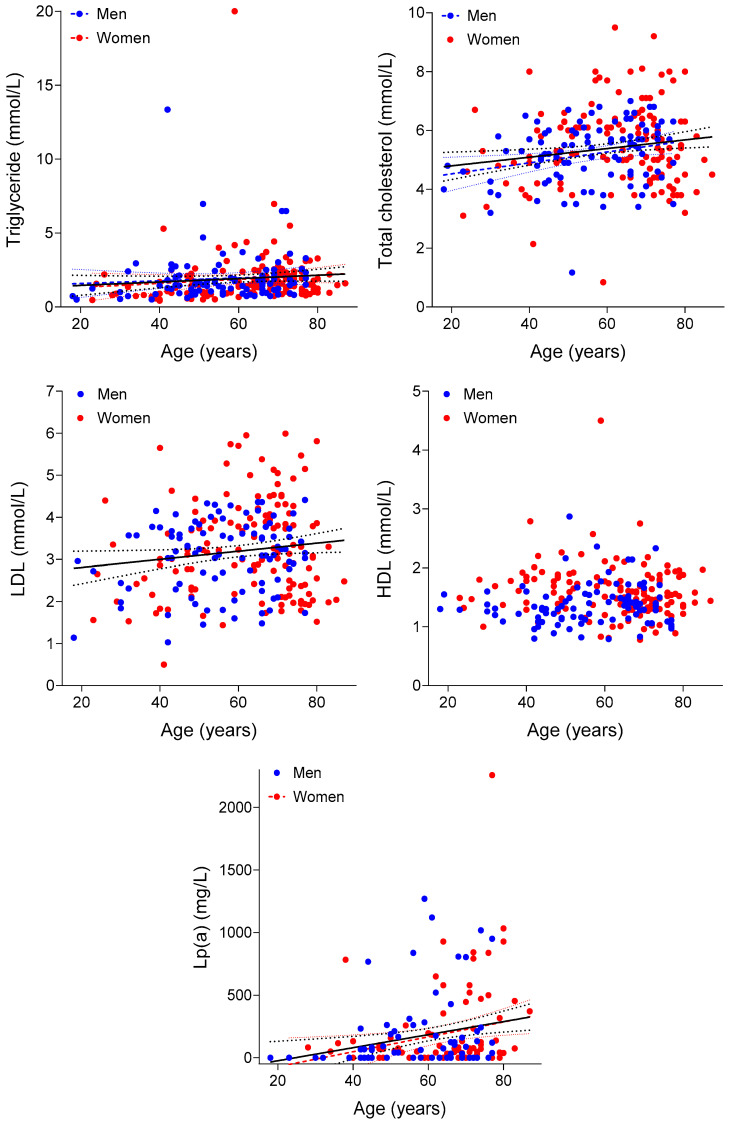
The correlation between the main serum lipid parameters (indicated in the axis *y* titles) and the age of our patients involved in this study (with chronic, idiopathic, and subjective tinnitus). A statistically significant correlation is indicated by the best-fit straight line and its 95% confidence bands obtained with linear regression using the whole data set (continuous line and dotted curves in black), the data set of women (dashed line and dotted curves in red), or the data set of men (dashed line and dotted curves in blue). The significant correlation of the gender-specific data is also indicated by the red or blue dashed lines crossing the red or blue points denoting the gender on the panels.

Finally, regarding the major serum lipid parameters (triglyceride, total cholesterol, LDL-C, HDL-C, and Lp(a)), except for HDL-C, all showed a statistically significant correlation with the age of the patients (Figure 3; Table 11). In some cases, this correlation remained significant even after the breakdown by gender, despite the decrease in sample size (Figure 3). However, in all cases, the observed correlation was weaker than expected (Figure 3).

## 4. Discussion

Over the past decades, numerous studies have explored the relationship between tinnitus and lipid metabolism [33]. Many researchers have confirmed a connection between abnormal blood lipid levels and tinnitus [16,20,21,28,34]. Among these, certain authors have linked elevated triglyceride levels, while others have associated elevated cholesterol, particularly elevated LDL levels, with tinnitus. According to our findings, none of the analyzed parameters exhibited an exceptionally high abnormality rate that would suggest a particularly prominent role. Elevated serum triglyceride levels and LDL levels were observed at similar rates (approximately 40%) throughout the study group, while serum cholesterol levels were elevated in a greater proportion of cases (57.1%) compared to the other important key parameters. Conversely, some researchers have rejected or questioned this relationship, as their results did not support this association or they did not find it to be statistically significant [22,23,35,36]. Other authors have examined how the altered lipid metabolism can influence the function of the inner ear at a cellular level, using animal models, seeking links between tinnitus, hearing loss, and abnormal lipid metabolism. Their results suggest that elevated lipid levels cause direct lipid accumulation in the cell membranes of the outer hair cells, thereby increasing the membrane rigidity and reducing the electromotility of the hair cells [37,38]. Moreover, considering the cochlea as an “end-organ” based on its blood supply, abnormalities in lipid metabolism can easily lead to cochlear hypoxia and damage, thereby harming both the hair cells and the stria vascularis [20,29,36]. Thus, the relevant literature presents conflicting data regarding the relationship between tinnitus and lipid metabolism. However, these ideas do not account for the particular forms of tinnitus that develop instead of normal hearing. It is worth noting that pure-tone audiometry typically evaluates hearing thresholds within the 125–8000 Hz range, as defined by the American National Standards Institute (ANSI), to classify normal hearing. However, the human ear’s hearing range extends from 20 to 20,000 Hz. This raises the possibility that in these cases, hearing loss may be present in the 8000–20,000 Hz range. Hearing impairment at these high frequencies can be assessed using high-frequency audiometry. This type of examination requires specialized equipment, which is not widely available, resulting in relatively limited data on this frequency range.

The literature contains limited data on the efficacy of treatments for dyslipidaemia in managing tinnitus. However, studies have reported the beneficial effects of a low-cholesterol diet and statin therapy in tinnitus associated with noise-induced hearing loss. Although our study did not aim to investigate therapeutic options, it is worth noting that clinically significant, measurable tinnitus was present in 7.79% of patients in our cohort who had previously diagnosed and treated dyslipidaemia. Thus, tinnitus persisted despite lipid-lowering therapy in these cases. Additionally, among treated dyslipidaemic patients, we found a high prevalence (77.78%) of abnormal serum lipid levels during our assessments.

In our present study, we compared the prevalence of the different abnormalities in the lipid metabolism in our patients with chronic subjective idiopathic tinnitus (CSIT) (receiving intravenous rheologic therapy at the Department of Otolaryngology and Head and Neck Surgery, Clinical Center, University of Debrecen) to the official prevalence data of dyslipidaemia in the general adult Hungarian population (according to the HCSO). To counteract the distorting effect of the co-increase in both conditions with age, we also examined the relationship between CSIT and lipid metabolism by age groups.

Our results indicate that in comparison with the general adult Hungarian population, the prevalence of abnormal lipid metabolism is significantly higher among our patients with CSIT, investigating it both overall and by each age groups. This association between CSIT and lipid abnormalities also highlights the key importance of the often asymptomatic dyslipidaemia. Based on the findings of the present investigation, it can be stated that lipid profile examination should play a significant role in the diagnostic protocol for patients with CSIT.

Limitations: An age- and gender-matched control group was not utilized and there were a small number of participants in each age group. Additionally, serum lipid levels were only classified as normal or abnormal. Secondly, detailed audiological data—such as pure-tone audiometry results, the frequency, and the intensity of tinnitus—were not analysed or compared. Furthermore, the outcomes of the Tinnitus Handicap Inventory (THI) questionnaires were also not elaborated upon or analysed for comparison.

## 5. Conclusions

The severity of tinnitus is currently evaluated based on the burden it imposes on the patient and its negative impact on their quality of life. However, our findings suggest that the importance of tinnitus is not only determined by the current distress it causes but also by its potential role as an indicator of underlying, asymptomatic, yet critical risk factors, such as dyslipidaemia. This is mainly attributed to the asymptomatic nature of dyslipidaemia, highlighting the importance of laboratory assays, patient education, and vigilance regarding dyslipidaemia. In light of this, CSIT should be regarded as more than a simple symptom that may impair the patient’s quality of life. Considering the relationship between CSIT and dyslipidaemia, recommending lipidological evaluation for patients suffering from CSIT and informing patients and their healthcare providers that tinnitus could be a symptom of dyslipidaemia could be of importance, even at a young age. This approach can reduce mortality caused by ASCVD, acting as the first protective bastion against harmful and life-threatening lipid-related issues.

## Figures and Tables

**Table 1:** Results of pure tone audiometry (PTA).

Type of Audiological Examination				Results					Patients Number		
**Pure-tone Audiometry**											
				Normal hearing					51 (22.1%)		
				Sensorineural hearing loss (pure tone threshold > 25 dB HL at any frequency)					180 (77.9%)		
**Characteristics of Tinnitus**											
Laterality				Unilateral					116 (50.2%)		
				Right-sided					44 (37.9%)		
				Left-sided					72 (62.1%)		
				Bilateral					115 (49.8%)		
**Frequency (Hz/Patient Numbers)**											
125	250	500	750	1000	1500	2000	3000	4000	6000	8000	12,000
7	7	5	2	16	3	9	11	39	16	113	3
**Intensity**											
				Right ear					10–105 dB HL (average: 51.9 dB HL, SD ±22)		
				Left ear					10–105 dB HL (average: 53.1 dB HL, SD ±22.2)		

**Table 2:** The occurrence of the previously known dyslipidaemia in our whole clinical sample and the subgroups formed according to the age classification of HCSO.

Subgroups by Age	18–24	25–34	35–44	45–54	55–64	65–74	75–	Total
Known dyslipidaemia	0	0	0	1	3	6	8	18

**Table 3:** The occurrence of hypertriglyceridemia in our whole clinical sample and the subgroups formed according to the age classification of HCSO. *: including the pathological triglyceride levels.

Subgroups by Age	18–24	25–34	35–44	45–54	55–64	65–74	75–	Total
Abnormal value (patient number *)	1	2	7	11	17	41	13	92
Isolated elevation of triglycerides	-	-	3	4	2	2	-	-
Occurrence of abnormal values (%)	11.1%	20.0%	28.0%	27.5%	41.5%	51.8%	36.7%	39.8%

**Table 4:** The occurrence of hypercholesterolaemia in our whole clinical sample and the subgroups formed according to the age classification of HCSO. *: including the pathological triglyceride levels.

Subgroups by Age	18–24	25–34	35–44	45–54	55–64	65–74	75–	Total
Abnormal value (patient number *)	1	3	14	22	29	52	11	132
Occurrence of abnormal values (%)	16.7%	30%	56%	55%	70.7%	65.8%	36.7%	57.1%

**Table 5:** The occurrence of increased LDL-C levels in our whole clinical sample and the subgroups formed according to the age classification of HCSO. *: including the pathological triglyceride levels.

Subgroups by Age	18–24	25–34	35–44	45–54	55–64	65–74	75–	Total
Increased value (patient number *)	2	2	10	16	25	35	7	97
Occurrence of abnormal values (%)	33.3%	20%	40%	40%	61%	44.3%	23.3%	42%

**Table 6:** The occurrence of increased ApoB levels in our whole clinical sample and the subgroups formed according to the age classification of HCSO. *: including the pathological triglyceride levels.

Subgroups by Age	18–24	25–34	35–44	45–54	55–64	65–74	75–	Total
Patients with available data	3	5	17	25	33	56	24	163
Increased value (patient number *)	0	0	6	12	19	30	9	76
Occurrence of abnormal values (%)	0%	0%	35.3%	48%	57.6%	53.6%	37.5%	46.6%

**Table 7:** The occurrence of decreased HDL-C levels in our whole clinical sample and the subgroups formed according to the age classification of HCSO. *: including the pathological triglyceride levels.

Subgroups by Age	18–24	25–34	35–44	45–54	55–64	65–74	75–	Total
Decreased value (patient number *)	1	3	10	11	13	20	9	67
Occurrence of abnormal values (%)	16.7%	30%	40%	27.5%	31.7%	25.3%	30%	29%

**Table 8:** The occurrence of decreased ApoA levels in our whole clinical sample and the subgroups formed according to the age classification of HCSO. *: including the pathological triglyceride levels.

Subgroups by Age	18–24	25–34	35–44	45–54	55–64	65–74	75–	Total
Patients with available data	3	5	17	25	35	61	24	170
Decreased value (patient number *)	1	0	2	2	5	4	1	15
Occurrence of abnormal values (%)	33.3%	0%	11.8%	8%	14.3%	6.6%	4.2%	8.8%

**Table 9:** The occurrence of increased Lp(a) levels in our whole clinical sample and the subgroups formed according to the age classification of HCSO. *: including the pathological triglyceride levels.

Subgroups by Age	18–24	25–34	35–44	45–54	55–64	65–74	75–	Total
Patients with available data	2	4	17	22	33	56	24	158
Increased value (patient number *)	0	0	2	0	8	13	9	32
Occurrence of abnormal values (%)	0%	0%	11.8%	0%	24.2%	23.2%	37.5%	20.3%

**Table 10:** The occurrence of dyslipidaemia among our patients and in the whole adult Hungarian population (HCSO data).

Subgroups by Age (Years)	Our Sample (Patient Number)	Dyslipidaemia in Our Sample (Patient Number and %)	HCSO Data (%)
18–24 years	6	3 (50%)	0.5%
25–34 years	10	4 (40%)	1.7%
35–44 years	25	20 (80%)	6%
45–54 years	40	28 (70%)	14.2%
55–64 years	41	38 (92.7%)	27.9%
65–74 years	79	64 (81%)	36.9%
75–84 years	30	25 (83.3%)	42.8%
Total	231	181 (78.4%)	16.5%

**Table 11:** The results of correlation between the main serum lipid parameters and the age of our patients involved in this study (with chronic, idiopathic, and subjective tinnitus). r: correlation coefficient (according to Pearson or Spearman); CI: confidence interval.

	Triglyceride	Total Cholesterol	LDL	HDL	Lp(a)
Pearson r (CI)	-	0.17 (0.04–0.29)	0.14 (0.01–0.26)	-	-
Spearman r (CI)	0.25 (0.12–0.37)	-	-	0.023 (−0.11–0.16)	0.22 (0.06–0.37)
*p*-value	0.0001	0.0092	0.0395	0.7289	0.0058

## Data Availability

Data available on request due to ethical restrictions.
